# Theory-practice gap: Nursing students’ self-reported depth of understanding of bioscience and its relevance to clinical practice

**DOI:** 10.1371/journal.pone.0294319

**Published:** 2023-11-30

**Authors:** Bronwynne Rafferty, Katlego Mthimunye, Million Bimerew

**Affiliations:** 1 School of Nursing, Faculty of Community and Health Science, University of the Western Cape, Cape Town, Western Cape, South Africa; 2 Department of Nursing, Midwifery and Health, Faculty of Health and Life Sciences, Northumbria University, Newcastle Upon Tyne, United Kingdom; University of Oxford, UNITED KINGDOM

## Abstract

**Background:**

Bioscience subjects are essential as they allow nurses to have a clear understanding of the patient’s condition and ultimately allow them to provide appropriate and timeous care. However, these subjects remain a significant problem in the endeavour to produce highly competent nurses.

**Aim:**

The study aimed to investigate the nursing students’ self-reported depth of understanding of bioscience and its relevance to clinical practice.

**Methods:**

A quantitative research approach using a descriptive survey design was employed. The sample (n = 211) included second-, third- and fourth-year undergraduate nursing students. A three-part self-administered questionnaire was adapted and used to collect the data. Data were analysed using IBM Statistical Package for Social Sciences software version 25.0 (IBM SPSS-25). Descriptive statistics and Chi-squared test were performed to describe the relationship among the study variables.

**Results:**

Participants rated their understanding of the application of Human Biology (n = 86, 40.76%) and Pharmacology (n = 88, 41.71%) as good, while Physics (n = 80, 37.91%) and Chemistry (n = 85, 40.28%) were rated as adequate. Most participants rated Human Biology (n = 175, 83.73%) and Pharmacology (n = 181, 86.19%) as essential, while Physics (n = 129, 61.72%) and Chemistry (n = 133, 63.64%) were rated as relevant to clinical practice. Physics (n = 112, 60.54%; n = 95, 50.53%) and Chemistry (n = 126, 68.85%; n = 113 61.41%) were rated as not relevant to monitoring a patient’s heart rate and blood pressure. Participants’ perception of the relevance of Chemistry in monitoring a patient’s blood pressure was statistically significant (χ^2^ = 6.871 (df 2), p<0.05). Most participants (n = 57, 41.91%) performed at Task specific on Akinsanya’s Bionursing model, suggesting an overall understanding of the foundational concepts and principles of bioscience.

**Conclusion:**

The findings of the study provided evidence of the students’ self-reported depth of understanding and perception of the relevance of bioscience and indicate a need for more emphasis on the importance of bioscience integration in clinical practice.

## Introduction

Bioscience subjects are the core foundation of the nursing curricula, and they play a pivotal role in pre-registration nursing education [1]. Unarguably, the core objective of nursing education is to equip nurses with the necessary knowledge to contribute positively towards high-quality patient care [2, 3]. Likewise, knowledge in biosciences has resulted in an increase in nurses’ confidence to carry out their roles and challenge practice if required [4]. A firm understanding and application of basic scientific concepts are vital in developing competent nurses. Thus, it is imperative to gain the necessary knowledge, level of clinical competency and complex decision-making skills needed in the current highly technical and rapidly evolving healthcare environment [5].

Bioscience is the scientific foundation for nursing practice [6]. Good biological knowledge guides good patient care [7, 8]. Whilst nursing education has progressed in recent times, the role of the nurse has expanded as nurses are expected to be independent [8]. Employers expect them to not only possess a specialised knowledge base but to also competently apply this knowledge to solve multifaceted health problems through effective approaches to care [9]. Thus, the ability of registered nurses (RNs) to progressively apply their bioscience-based interpretation skills to recognise cases of concern is paramount and can never be over-emphasised [2].

As with many developing countries, nurses in South Africa are required to practice independently and autonomously. This is exacerbated by the fact that graduate nurses in South Africa are required to work in rural areas [10] where there is restricted access to medical doctors [11]. The Department of Health [12] reported that rural areas in South Africa contain 43.6% of the population but are assisted by only 12% of medical doctors and 19% of nurses. The obvious imbalanced nurse-patient ratio further highlights the importance of ensuring that graduate nurses’ decision-making processes are informed by science and that function optimally within their scope of practice.

The South African Nursing Council (SANC) Regulation 425 of the Nursing Act of 2005 as amended stipulates that General Nursing Science is a compulsory module in the nursing curriculum. It forms the foundation of nursing science by promoting an understanding and application of bioscience modules [13]. The regulation required the incorporation of bioscience with major nursing modules across the pre-registration nursing programmes. However, the means of integration is not documented leading to fewer hours being assigned to bioscience compared to other nursing modules, thereby demoting bioscience to the status of subordinate subjects [14]. This phenomenon has adversely contributed to the declining and devaluing of bioscience modules in the preregistration nursing curriculum [15, 16]. The same argument has been echoed in a survey conducted in 2015 to examine the biosciences element of preregistration nursing programmes in tertiary institutions throughout the UK [8]. This study revealed that nursing students perceived bioscience content in the nursing curriculum as insufficient [8].

Although the degree of difficulty and complexity of a module is subjective, many authors agree that nursing students generally perceive bioscience subjects to be difficult to learn and understand [3, 5, 17, 18]. Two recent South African studies reflect students’ poor performance in bioscience [11, 13]. In a study conducted by Rafferty et al. [11] at a nursing college in the Western Cape, South Africa, approximately 50% of nursing students failed to meet the minimum pass mark in the bioscience primary examination. This was substantiated by an unpublished masters study conducted by Mthimunye [19] who found that 52.21% of the second-year nursing students in 2012–2013 who hold a National Senior Certificate failed to complete bioscience modules at the first attempt. Bioscience modules are now stigmatized and are regarded as “killer modules” by nursing students [20].

The findings of an explanatory sequential mixed methods study conducted by Fell et al. [4] suggest that the importance of biosciences in nursing practice is not emphasised enough during the first- and second year of preregistration nursing. At the university understudy, Bioscience modules for preregistration nursing curriculum include Human Biology, Physics, Chemistry and Pharmacology. The modules are taught predominantly during the first 2 years of the preregistration nursing programme of the R425 programme. Bioscience modules aim to equip nursing students with useful knowledge regarding the Anatomy, Physiology and Pathophysiology of the human body, the causes of these changes and the indications or mechanism of actions of various medications that are used as treatment [21]. Comprehension of these crucial bioscience concepts permits nursing students to express the reasons for providing care [21].

The poor performance in bioscience could be attributed to the failure to bridge the gap that exists between nursing theory and clinical practice [22, 23] and it is evident that this gap needs to be strengthened early [24]. Nursing students find it challenging to correlate the relevance of biosciences to clinical practice. Subsequently, they experience anxiety and have poor confidence in appropriately applying concepts to patient conditions [25]. Students learning in clinical practice may also be affected by the increased workload in placements [26] and a negative ward culture that devalues the importance of biosciences in the clinical placement environments [4]. The clinical environment is arguably the best environment for the integration of bioscience knowledge into clinical practice as it gives students a realistic environment in which to apply bioscience theory to practice [11]. However, Bakon et al. [17] noted that nursing students find it challenging to apply bioscience knowledge in clinical practice. Thus, it was deemed necessary to investigate the nursing students’ self-reported depth of understanding of bioscience and its relevance to clinical practice. The following objectives were developed and are reported in this paper: (1) To establish the nursing students’ self-reported knowledge of bioscience (yes, no); (2) to establish the nursing students’ self-reported depth of understanding of bioscience (superficial, adequate, deep) and (3) to determine the nursing students’ self-rated perceptions of the relevance of bioscience to clinical practice (not relevant, relevant, essential).

### Conceptual framework

Akinsanya’s [6] Bionursing conceptual model was used to guide the analysis of the descriptive items of the questionnaire (items 12.1 and 12.2). This theoretical framework was developed for the nursing curriculum as a way of strengthening the Life Science in nursing curricula [6]. Akinsanya [6] suggested four levels of task performance which note the depth of knowledge and understanding of the Life Sciences on which nursing care depends. The levels reflected upon are task operational, task-specific, task contextual as well as personal and professional development.

## Materials and methods

### Study design

This research employed a quantitative research approach with a descriptive survey design [27]. A three-part self-administered questionnaire was completed by preregistration nursing students at a selected university.

### Research setting

The study was conducted at a university in the Western Cape province of South Africa. The university offers a range of nursing programmes accredited by SANC the Education and Training Quality Assurer (ETQA) as well as the South African Qualifications Authority (SAQA). The pre-registration nursing programme which constitutes 50% theory, and 50% clinical practice was the focus of the study. To complete the programme, nursing students are required to complete 4000 hours of clinical placements. These hours are distributed across four specialised areas of nursing as follows: 1900 hours for General Nursing Science (GNS), 500 hours for Community Nursing Science (CNS), 600 hours for Psychiatric Nursing Science (PNS) and 1000 for Midwifery Nursing Science (MNS). Completion of these hours is monitored under the strict supervision of clinical mentors and registered nurses [28].

### Population and sample

The target population (N = 758) comprised of 2^nd^ 3^rd^ and final year preregistration nursing students who have undertaken bioscience-related modules before or during the 2020 academic year. Using the sample size equation n = (p) (1−p) (Z)^2^/e^2^ and n_a_ = n/(1 + (n—1)/N) the adjusted sample size (n_a_ = 255) was calculated based on 95% confidence level and 5% margin of error. Simple random sampling was used as the sampling strategy for this study. Elements are at random from a sampling frame [27]. Following the selection of the sample, the researchers together with two research assistants collected the data between September and October 2020.

### Survey design and validation

A three-part items self-reported survey was adapted from the surveys available in the literature [11, 29]. The original survey was developed at determining the depth of registered nurses’ knowledge and use of biosciences [29]. The questionnaire was later adapted by Rafferty et al. [11] to establish the fourth-year student nurses’ self-reported knowledge and understanding of the biosciences and their perception of the relevance thereof to clinical practice. Reliability was tested by Rafferty et al. [11] by using the Cohen’s Kappa test which revealed that raters were all above 0.6. This indicates agreement between the raters [30]. Permission to use the questionnaire was granted and was adapted to suit the study context. The bioscience modules on the original questionnaire were replaced with the bioscience modules from the preregistration nursing programme at the university under study.

The adapted survey gathered data on the participants’ demographics as well as their knowledge and understanding of bioscience and its relevance to clinical practice. The questionnaire consisted of 3 parts and a total of 36 items. Part 1 of the questionnaire gathered data pertaining to the participant’s demographics as well as the self-reported depth of understanding of bioscience modules (superficial/adequate/deep) and their application (very poor/ poor/adequate/good/very good) to clinical practice. Part 2 of the questionnaire required participants to describe a critical incident from their experience and explain how their bioscience knowledge and understanding of bioscience ensured a quality patient outcome. For part 3—Picture interpretation of nursing interventions, pictures portraying three clinical scenarios were presented to the participants. The scenarios included i) a nurse monitoring a patient’s heart rate; ii) a nurse monitoring a patient’s blood pressure and iii) a nurse motoring a patient’s temperature. Throughout the undergraduate years of training, the most frequently assigned task for nursing students is to monitor a patient’s physiological vital signs in numerous clinical disciplines [31]. Nurses are required to determine the patient’s condition by means of measuring and interpreting patient’s vital signs. Participants were required to indicate whether knowledge and understanding of any or all bioscience modules are relevant or not relevant for each of the given scenarios.

Content validity was ensured by the research supervisors (experts in learning, teaching, and assessment in nursing education) and a statistician [27]. The questionnaire was refined, and grammatical errors were corrected to enhance the accurateness of the questions. A pre-test of the questionnaire was done on 12 preregistration nursing students (selected via convenience sampling) who met the inclusion criteria to ensure that the questions were coherent [32]. The participants fully understood the questions they were being asked, and they answered appropriately. Cronbach’s alpha is a measure used to assess the reliability, or internal consistency, of a set of scale or test items [33]. An internal consistency test revealed a moderate but acceptable Cronbach’s alpha coefficient of less than 0.8 (α < 0.8) [34]. It was, however, noted that the Likert-scale rating was inconsistent for items 7.1 to 7.4. The inconsistencies (deep understanding = 3, adequate understanding = 2, superficial understanding = 1) were corrected in by adopting the following scale: deep understanding = 1; adequate understanding = 2; superficial understanding = 3. Finally, after the adjustments, the internal consistency test revealed an acceptable and improved Cronbach’s alpha coefficient of α = 0.843 [35]. The pre-test questionnaires were excluded from the main analysis of the study as changes were made to the questionnaire.

### Data analysis

Data were analysed using IBM Statistical Package for Social Sciences version 25 (IBM SPSS-25). Pairwise deletion was employed to manage any missing data [36].

Descriptive statistics were performed by analysing the frequency of responses, measures of central tendency, percentage, and proportions. For the items relating to the critical incident, participants were asked to describe one incident which fitted into one of four categories in Akinsanya’s [6] Bionursing Framework (task operational, task-specific, task-contextual, and personal and professional development). Responses were analysed using frequency and proportion. Inferential statistics, and Chi-squared tests were performed to test relationships between year level and any of the indicator variables. Four pre-listed bioscience modules were indicator variables for the ordinal 3-point rating scale, ordinal 5-point rating scale and binary questions. The p-value for significance was set at p < 0.05 [37].

### Ethical considerations

Ethical clearance (Registration No. HS20/2/4) was sought from the University’s Humanities and Social Sciences Research Ethics Committee. The registrar, Head of Department (HoD), portfolio leads, and year-level coordinators granted permission for the study to be conducted. Informed consent was obtained prior to data collection and all participants were informed about their right to autonomy. The principles of beneficence, non-maleficence anonymity and confidentiality were ensured throughout the study [27]. Any form of identification such as students’ names, contact details, student numbers and university names are withheld to safeguard the study participants. All data were kept in a safe and secure place and could be made available upon request.

## Results

### Participants

A total of 211 (82.74%) out of 255 participants completed the survey. Of the 255 participants, 76 (36.19%) were in their second year of study, while 97 (46.19%) and 37 (17.62%) were in their third and final year respectively. There was one (0.47%) missing data. The analysis of the demographic data revealed that the majority of students were female (167; 79.15%) while the male students were the minority (44; 20.85%). The youngest participant was 18 years old and the oldest was 44 years old. The median age of the participants was 21 years while the mean age was 22 with a standard deviation (SD) of 3.508. According to the data, 208 (98.58%) participants had no previous nursing qualifications, while only 3 (1.42%) participants reported having a previous nursing qualification. Similarly, 206 (97.63%) participants did not possess any previous nursing work experience, while 5 (2.37%) participants indicated having previous experience in nursing work.

### Participants’ self-reported depth of understanding of biosciences

Participants rated their understanding of bioscience modules (Part 1) using a 3-point Likert-scale ranging from superficial understanding to deep understanding. The overall results revealed that the majority of responses (447/838, 53.34%, 6 missing data) participants have an adequate understanding of all bioscience modules. A deep understanding was reported for Human Biology (n = 81, 38.76%). For Physics, Chemistry and Pharmacology understanding was reported as superficial (n = 61, 29.05%; n = 63, 30%; n = 43, 20.57%), respectively (Table 1). The Chi-squared test showed no significant association between participants’ understanding of the bioscience modules and their year level (p>0.05). Therefore, these results indicate that participants’ understanding of bioscience modules is not dependent on their year level.

**Table 1:** Participants’ self-reported level of understanding of bioscience.

Subject	Superficial understanding	Adequate understanding	Deep understanding	χ^2^ (df) = 4	p-value
	n (%)	n (%)	n (%)		
**Human biology (n = 209)**	21 (10.05%)	107 (51.20%)	81 (38.76%)	8.100	.088
**Physics (n = 210)**	61 (29.05%)	103 (49.05%)	46 (21.9%)	6.118	.190
**Chemistry (n = 210)**	63 (30%)	103 (49.05%)	44 (20.95%)	7.679	.104
**Pharmacology (n = 209)**	43 (20.57%)	134 (64.11%)	32 (15.31%)	5.857	.210

### Participants’ self-reported understanding of the application of bioscience to clinical practice

A 5-point Likert-scale was used to investigate the participants’ understanding of the application of the bioscience modules to clinical practice (Part 1). The options were as follows: Very poor; Poor; Adequate; Good and Very good. The majority of the responses (n = 296/844, 35.07% and n = 188/844, 22.27%) across all four bioscience modules indicated a good to very good application of bioscience knowledge to clinical practice respectively, while n = 257/844 (30.45%) indicated adequate understanding, n = 79/844 (9.36%) indicated poor understanding and n = 24 (2.84%) indicated very poor understanding. The Chi-squared test shows no significant association between participants’ understanding of the application of bioscience modules to practice and their year level (p>0.05). As a result, these results suggests that participants’ understanding of bioscience modules is not dependent on their year level (Table 2).

**Table 2:** Participants’ self-reported understanding of the application of bioscience theory to practice.

Subject	Very poor	Poor	Adequate	Good	Very good	χ^2^ (df) = 8	p-value
	n (%)	n (%)	n (%)	n (%)	n (%)		
**Human biology (n = 211)**	1 (0.47%)	2 (0.95%)	38 (18.01%)	86 (40.76%)	84 (39.81%)	5.248	.731
**Physics (n = 211)**	9 (4.27%)	36 (17.06%)	80 (37.91%)	63 (29.86%)	23 (10.90%)	3.955	.861
**Chemistry (n = 211)**	10 (4.74%)	31 (14.69%)	85 (40.28%)	59 (27.96%)	26 (12.32%)	5.127	.744
**Pharmacology (n = 211)**	4 (1.90%)	10 (4.74%)	54 (25,59%)	88 (41.71%)	55 (26.07%)	5.427	.711

### Participants’ self-reported perception of the relevance of bioscience in clinical practice

Participants were required to rate the relevance of the bioscience subjects to clinical practice using a 3-point Likert scale ranging from not relevant, relevant, to essential. Table 3 indicates that most participants found the bioscience subjects relevant and essential to clinical practice. Pharmacology and Human biology were considered to be essential for clinical practice (n = 181, 86.19%; 175, 83.73% respectively), this was followed by participants rating Physics and Chemistry as relevant (n = 129, 61.72%; n = 133, 63.64% respectively) but not essential. Overall, participants (42.58) perceive Physics and Chemistry as not relevant to clinical practice. The Chi-squared test results indicates that the participants’ perception of the relevance of the bioscience modules to nursing practice is not associated with their year level as p>0.05.

**Table 3:** Participants’ self-rated perception of bioscience modules relevance to nursing practice.

Subject	Not relevant	Relevant	Essential	χ^2^ (df) = 4	p-value
	n (%)	n (%)	n (%)		
**Human biology (n = 209)**	3 (1.44%)	31 (14.83%)	175 (83.73%)	5.001	.287
**Physics (n = 209)**	51 (24.40%)	129 (61.72%)	29 (13.88%)	1.781	.776
**Chemistry (n = 209)**	38 (18.18%)	133 (63.64%)	38 (18.18%)	1.114	.892
**Pharmacology (n = 210)**	5 (2.38%)	24 (11.43%)	181 (86.19%)	3.623	.459

### Descriptions of critical incidents, student interventions and use of bioscience to improve patient outcomes

A total of 136/211 (64.4%) responses were noted during the analysis of which 129/136 (94.85%) were valid and analysed. The Akinsanya’s (1987) Bionursing conceptual model was employed to categorise and quantify participants’ level of performance as follows: (1) Task operational (lower level); (2) Task specific; (3) Task contextual; (4) Personal and Professional development (High level). The results revealed that 57 (41.91%) performed interventions as task level two indicating that the majority of participants have an understanding of the foundational concepts of biosciences such as terms and principles aimed at carrying out specific tasks. Thirty-nine percent (n = 54) performed at operational task level one while n = 16 (11.76%) and n = 2 (1.4%) performed at level-three and -four respectively. Table 4 summarises the findings of the participant’s description of a critical incident.

**Table 4:** Participants’ description of a critical incident example.

Akinsanya’s (1987) Conceptual framework [n = 129]			
**Task level (Number of responses)**	**Criteria**	**Example of incident**	**Perception of knowledge needed (example)**
**Task operational (54)**	At this level of performance, the activities done by nurses do not require a specific depth of knowledge of the biosciences	“I was working at traumas when we received a patient that was stabbed on the thigh and there was a lot of bleeding.” (P133).	Human biology- apply pressure to stop the bleeding to form blood clotting
**Task specific (57)**	The nurse is required to have an understanding of the foundational concepts of bioscience such as terms and principles intended for performing particular tasks.	“Patient had to be given an injection.” (P13)	Implied but not stated: Anatomy Implied but not stated: Pharmacology
**Task contextual (16)**	The nursing activities hinge on accurate knowledge of life sciences for patient safety. This level requires the nurse to have a deep understanding and can apply the concepts and principles of bioscience to a particular task.	“Patient came to our ward complaining of diarrhoea, vomiting and abdominal pain.” (P21)	Human biology-locating pain Physics-accurate dosage calculation of medication Chemistry constituents of electrolyte fluids Pharmacology-medication to give to the patient to reduce pain
**Personal and Professional development (2)**	The nurse is required to be capable of rationalizing all actions and be responsible for linking theory to practice	“When I was working my midwifery block, one of the patients was losing a lot of blood (post-partum haemorrhage) and I knew I had to give Oxytocin and Voluven to ensure the safety of the patient.” (P86)	No description Human biology Colloid needed for blood loss Pathophysiology: post-partum haemorrhage Pharmacology: Oxytocin given

P = Participant number

### Picture interpretation of nursing interventions: Perceptions of the relevance of bioscience modules for monitoring patient’s vital signs

Participants were required to rate the relevance of the bioscience modules in monitoring a patient’s heart rate, blood pressure and temperature as relevant or not relevant indicates that the majority of participants rated Human biology and Pharmacology were rated between 70.41% and 99.52% for relevance for monitoring heart rate, blood pressure and temperature. While Physics and Chemistry were considered to be relevant for monitoring temperature readings (55.91% and 55.85% respectively). Results of the participants self-rated perceptions of the relevance of bioscience modules for monitoring patient’s vital signs is shown in Table 5.

**Table 5:** Participants self-rated perceptions of relevance of bioscience to monitoring vital signs.

**Participants’ self-rated perception of the relevance of bioscience to monitoring a patient’s heart rate**					
Subject	**Relevant**		**Not relevant**	**χ**^**2**^ **(df) = 2**	**p-value**
	**n (%)**		**n (%)**		
Human Biology (n = 207)	206 (99.52%)		1 (0.48%)	4.745	.093
Physics (n = 185)	73 (39.46%)		112 (60.54%)	4.642	.098
Chemistry (n = 183)	57 (31.15%)		126 (68.85%)	2.7966	.247
Pharmacology (n = 196)	138 (70.41%)		58 (29.59%)	3.581	.167
**Participants’ self-rated perception of the relevance of bioscience to monitoring a patient’s blood pressure**					
Subject	**Relevant**		**Not relevant**	**χ**^**2**^ **(df) = 2**	**p-value**
	**n (%)**		**n (%)**		
Human Biology (n = 207)	206 (99.52%)		1 (0.48%)	1.151	.562
Physics (n = 188)	93 (49.47%)		95 (50.53%)	0.407	.816
Chemistry (n = 184)	71 (38.59%)		113 (61.41%)	8.871	.032
Pharmacology (n = 196)	149 (76.02%)		47 (23.98%)	4.129	.127
**Participants’ self-rated perception of the relevance of bioscience to monitoring a patient’s temperature.**					
**Subject**	**Relevant**	**Not relevant**		**χ**^**2**^ **(df) = 2**	**p-value**
	**n (%)**	**n (%)**			
Human Biology (n = 206)	200 (97.09%)	6 (2.91%)		1.189	.552
Physics (n = 186)	104 (55.91%)	82 (44.09%)		2.551	.279
Chemistry (n = 188)	105 (55.85%)	83 (44.15%)		4.491	.106
Pharmacology (n = 196)	145 (73.98%)	51 (26.02%)		2.935	.230

Physics and Chemistry are considered to be not relevant in monitoring a patient’s heart rate and blood pressure. For monitoring a patient’s heart rate, participants considered Physics (60.54%) and Chemistry (68.85%) as not relevant. Furthermore, for monitoring a patient’s blood pressure, the participants rated Physics (50.53%) and Chemistry (61.41%) as not relevant. A chi-square test was performed to determine if there is an association between the participant’s year level and their perception of the relevance/non-relevance of monitoring a patient’s heart rate, blood pressure and temperature. The results revealed a significant association (χ^2^ = 6.871 (df 2), p<0.05) between Chemistry for monitoring a patient’s blood pressure and the year level of participants. No statistical significance between the participants’ year level and Human Biology, Physics and Pharmacology as p > 0.05.

## Discussion

This study aimed to investigate the nursing students’ self-reported depth of understanding of bioscience and its relevance to clinical practice. The following key findings emerged from this study: i) participants reported an adequate understanding of all bioscience modules with Human biology receiving the highest rating for deep understanding and Chemistry having the highest rating for superficial understanding; ii) a good to very good application of Human Biology and Pharmacology to clinical practice was reported respectively, while application of Physics and Chemistry was reported as adequate; iii) When asked to describe a critical incident and intervention most participants seemed to perform at task operational and task specific which indicates they have an adequate understanding of bioscience principles and terms for performing specific tasks; iv) Human biology and Pharmacology were deemed to be relevant to clinical practice for monitoring a patient’s heart rate, blood pressure and temperature while Physics and Chemistry were deemed relevant only for monitoring temperature.

Bioscience is a pivotal component underpinning undergraduate nursing programmes [38]. In the current study, participants increasingly reported a deeper understanding of Human Biology and Pharmacology than Physics and Chemistry. The participants placed more importance on Human Biology and Pharmacology than on Physics and Chemistry. This could be explicated by previous knowledge of the participants as Life Sciences and Mathematics are compulsory subjects whilst Physical Science is a recommended subject for admission into the undergraduate nursing programme [39]. Furthermore, this result could be due to the explicit relationship between Human Biology and Pharmacology in nursing practice. In addition, if academics do not prioritize these modules, it is clear that the students will not see the need to gain more understanding of these modules.

The majority of participants were in their second and third years and ought to be functioning between task specific and task contextual where the demand for bioscience knowledge becomes greater. Notwithstanding, this study’s findings revealed that the students at the identified university perform at task operational and task specific on the Akinsanya’s [6] Conceptual framework suggesting that the population understudy is competent in performing basic nursing tasks and understanding basic scientific concepts while they lack accurate knowledge of life sciences for patient safety and are not capable of rationalizing all actions and be responsible for linking theory to practice. This lack of rationalisation indicates a poor knowledge of bioscience, poor integration, and poor decision-making skills. This may be explicated by majority of participants having no previous nursing qualification or work experience. This is further substantiated by Wangensteen et al. [40] who denotes that variables such as gender, educational level, work experience activities are predictors for nursing competence and work experience is a factor that impacts clinical competence [40, 41]. It is undeniable that nursing students find biosciences to be the most difficult subjects of their training and they find understanding and applying bioscience knowledge to be challenging [42–44]. However, it is important to note that bioscience knowledge is important for clinical decision-making that will maximize health outcomes, conducting a physiological assessment, performing clinical interventions and assessing treatment effectiveness [9, 45]. These skills are based on comprehensive bioscience knowledge and its association with clinical practice [46].

A clear understanding of Anatomy, Physiology and Biochemistry is vital for understanding the physiology of the human body as well as the pathophysiology of illness and diseases [44]. Likewise, registered nurses need to have a sufficient understanding of bioscience to underpin safe and effective clinical practice [15, 47]. Similar to the study of Rafferty et al. [11], the majority of participants in the present study reflected an adequate understanding of all bioscience modules and not deep, this may be due to inconsistency in the quality of support offered to students, the lack of learning opportunities and the low priority that bioscience is given in placement education [4] notwithstanding, that many students find science subjects difficult [48]. This study revealed that the participants understanding of Human Biology and Pharmacology was better than that of Physics and Chemistry, this could be related to the importance the lecturer gives the subjects and the relevance perceived by the students as over 20% have a superficial understanding of Physics, Chemistry.

Students in the study of Fell et al. [4] rated bioscience as essential, very important or important they clearly appreciated the need for bioscience knowledge in nursing. The students in the study of Barton et al. [46] demonstrated an understanding that bioscience education provides the foundation for safe clinical practice as a registered nurse. Similarly, this study found that nursing students value bioscience and perceived that it has a role in many aspects of nursing practice [26].

A factor for concern that arises from the current study is that the general population of participants perceived Physics and Chemistry as not relevant to nursing practice. Although a cross-sectional survey conducted by Birks et al. [50] suggests that registered nurses perceived subjects related to Anatomy, Physiology and Pathophysiology as highly relevant to nursing practice, the need to understand the principles of Chemistry which underpin these subjects is often overlooked [49]. This may be accounted for by academics not giving these subjects the necessary attention. In an Australian cross-sectional survey conducted by Birks et al. [50] with 30 academics who teach science to nursing students, the results of the study revealed that academics rated Microbiology, Chemistry and Physics as a moderate priority, and topics related to the biomechanics of movement were given a low ranking. Thus, if academics do not prioritize these subjects, it is clear that the students will not see the need to gain more knowledge in these modules.

This clear gap between Physics and Chemistry and how it underpins clinical practice may be the key to why more students reported these subjects as less relevant to nursing practice as compared to Human Biology and Pharmacology. In addition, students’ attitudes toward the value of science are constantly changing and when students find science challenging this leads to undesirable levels of anxiety which further perpetuate their negative perception of bioscience modules [42] resulting in them perceiving it as less valuable in nursing practice [48]. Although a fair amount of anxiety can be motivating (Deshpande and Kawane 1982 as cited in Cooper et al. [51]), high levels of anxiety can undermine the learning process and lead to irreversible negative perception towards the content that needs to be learned. Students who perceive learning content as anxiety-provoking often employs a surface approach to learning [52] which could explain the inability to comprehend the essential role some bioscience modules play in nursing practice.

Students with lower expectations and self-efficacy cannot see the relevance of bioscience to practice [3]. If nursing students disregard the importance and relevance of bioscience to their future role it might have consequences for the standard of their practice [11] and patient safety.

It is evident that the bioscience content of the undergraduate curriculum needs to be integrated with nursing practice in order for the students to be prepared and confident for practice [53]. Students emphasised that bioscience should be prioritised in their undergraduate nursing education to equip them with the bioscience knowledge and understanding to confidently take on their roles as nurses [4].

However, this integration may be difficult as biosciences in nursing education are often taught as separate disciplines by lecturers at many universities [42] and can form educational challenges regarding their relevance to nursing practice [44]. Bioscience lecturers may never have been exposed to the clinical environment and this lack of context can lead to the student’s poor integration of bioscience theory into the clinical setting [42]. This was echoed in the findings in a study conducted by Mthimunye et al. [20] revealed that educators described a lack of synchronicity in the programme modules, and this adversely influences consistent learning. They noted that because modules are taught separately, students are incapable of comprehending the association between programme modules.

Nurse educators should investigate ways to improve the integration of bioscience into nursing practice. Students emphasised that bioscience should be prioritised in their undergraduate nursing education to equip them with the bioscience knowledge and understanding to confidently take on their roles as nurses [4]. This can be done through a collaborative approach involving nursing educators working with bioscience lecturers (more specifically Physics and Chemistry) to provide the best solution for this problem [8]. Alternatively, nurse educators should teach bioscience subjects to nursing students to facilitate their knowledge, understanding and application to nursing practice. The academic staff need to create closer associations with practice so that learners can instantly recognize the relevance of bioscience content and the practice placement reality [44].

### Conclusions

This study offers information on the students’ self-reported depth of understanding of biosciences. The study also indicates the students’ self-reported perception of the relevance of biosciences in nursing practice. Study findings indicate that students are more confident in their knowledge and understanding of Human Biology and Pharmacology bioscience modules and less with Physics and Chemistry. They have shown less interest in Physics and Chemistry and have clearly indicated that it is not relevant to nursing practice as Human Biology and Pharmacology. The integration of these modules into nursing practice should be made clear for students to understand their relevance and improve their knowledge and understanding of the modules.

### Study strengths and limitations

As far as the authors are aware, following the study of Rafferty et al. [11] this is the first study in SA that reported on 2^nd^, 3^rd^ and final year nursing students’ self-reported depth of understanding of bioscience and its relevance to clinical practice. The research instrument of this study when tested using Cronbach’s alpha demonstrated high values (α = 0.843) indicating the reliability of the research instrument. The target population was not met due to the impact and unprecedented circumstances of the COVID-19 pandemic: the researcher had limited access to students due to protocols that were put in place to prevent the spread of the virus. Lastly, the research was only performed at one Higher Education Institution, therefore results should be generalised with caution. In spite of these limitations, the implications of our findings will be of interest to nurse clinicians, nurse educators and researchers.

### Implications for future research

The authors recommend further research on the subject to assess where improvement of the curriculum is needed. We recommend that similar studies should be conducted at other nursing schools with the aim of providing a detailed examination of self-reported depth of understanding of bioscience and its relevance to clinical practice across all the year levels of the nursing programme. It would also be beneficial to conduct a follow-up qualitative study aiming at exploring the in-depth understanding of the difficulties students experience with bioscience learning. Furthermore, we recommend conducting further research on how bioscience lecturers can make a clear link between bioscience and nursing practice particularly for Physics and Chemistry. Studies should be conducted on the measures that need to be taken by clinical areas and academics to bridge the theory-practice gap as well as innovative methodologies to enhance the learning and teaching of bioscience in nursing education.

### Implication for nursing practice

Clinical placements provide an ideal environment in which students could link bioscience theory and practice. It provides the ideal environment to integrate bioscience into clinical decision-making [4] and in turn confidently undertake their role as nurses providing quality care and positively influencing health outcomes. In addition, working with suitably experienced registered nurses could help to reinforce learning, bridge the theory-practice gap, and improve skills; however, this requires that the registered nurses are confident in their understanding of bioscience [54].

### Implication for nursing education

Nurse educators should investigate ways to improve the integration of bioscience into nursing practice. In addition, the academic staff should relate their content to clinical practice in order for students to immediately recognise the relevance of bioscience content and practice placement Jensen et al. [44]. This can be achieved through collaboration between nurse educators and bioscience lecturers (especially Physics and Chemistry) to provide the best resolution for this problem [8]. Alternatively, nurse educators should teach bioscience subjects to nursing students to facilitate their knowledge, understanding and application to nursing practice.

In a retrospective quantitative study conducted by Mortimer-Jones et al. [55] at an Australian university, results illustrated that the nursification (the active association of a subject with nursing theory and practice) motivated students, inspired them to learn and enabled effective learning. Results of a more recent study conducted by Owens [56] involving the delivery of a five-day pre-nursing bioscience and study skills intervention illustrated that participants described having a higher self-efficacy, their emotions and well-being were supported, and the intervention also provided an opportunity to see the association between bioscience and clinical practice.

McVicar et al. [54] maintained that learning outside the practice environment is vital; therefore, examples from practice should be introduced into the classroom environment. Furthermore, the implementation of bioscience learning or workshops during the final year of the undergraduate programme would enhance knowledge before students become registered nurses.

Innovative teaching methodologies such as Project-based learning and inquiry-based learning could be used as a pedagogical approach to teaching bioscience to nursing students. Project-based learning uses real-world scenarios, challenges, and problems to involve students in critical thinking, problem solving, teamwork and self-management [57] while Inquiry based learning exposed the students to real-life environment whereby students can learn specific topics actively by participating in research related to problems which occur in the real world [58]. These methodologies allow for the integration of the teaching process and practice [59] therefore resulting in further integration of knowledge and skills acquired in diverse courses [60] such as chemistry and physics.
